# A prospective study of iron status in exclusively breastfed term infants up to 6 months of age

**DOI:** 10.1186/1746-4358-3-3

**Published:** 2008-03-01

**Authors:** Shashi Raj, MMA Faridi, Usha Rusia, Om Singh

**Affiliations:** 1 Division of Neonatology, Department of Pediatrics, University College of Medical Sciences, Delhi, India; 2 Department of Pathology, University College of Medical Sciences, Delhi, India; 3 Immunoendocrinology Laboratory, National Institute of Immunology, New Delhi, India

## Abstract

**Background:**

Can exclusive breastfeeding until six months of age maintain optimum iron status in term babies? We evaluated iron status of exclusively breastfed term infants in relation to breast milk iron and lactoferrin.

**Methods:**

In this prospective study in Delhi, India, during the period 2003–2004 normally delivered babies of non-anemic [(Hemoglobin (Hb) = 11 g/dl, n = 68] and anemic (Hb 7 – 10.9 g/dl, n = 61) mothers were followed until 6 months of age. Iron parameters were measured in the cord blood at 14 weeks and 6 months. Breast milk iron and lactoferrin were measured at the same intervals.

**Results:**

Iron parameters in babies of both groups were within normal limits at birth, 14 weeks and 6 months. Mean breast milk iron and lactoferrin in non-anemic (day 1: 0.89, 6 months: 0.26 mg/l; day 1: 12.02, 6 months: 5.85 mg/ml) and anemic mothers (day 1: 0.86, 6 months: 0.27 mg/l; day 1: 12.91, 6 months: 6.37 mg/ml) were not different on day one or at other times. No relationship was found between breast milk iron, lactoferrin and iron status of the babies.

**Conclusion:**

Exclusively breastfed infants of non-anemic and anemic mothers did not develop iron deficiency or iron deficiency anemia by six months of age.

## Background

The World Health Organization (WHO) and American Academy of Pediatrics unequivocally recommend that exclusive breastfeeding is the ideal nutrition for infants and is sufficient to support optimal growth for the first six months of life [1,2]. However, there is controversy about the adequacy of breast milk in maintaining optimum iron status of exclusively breastfed babies. A WHO committee expressed concern that some exclusively breastfed infants may become iron deficient [1]. Glader recommended that infants exclusively breastfed should receive iron supplementation from four months of age [3]. Calvo et al. evaluated the iron and nutritional status of exclusively breastfed infants for a prolonged period in relation to their growth rate and dietary changes and recommended that breastfed infants should be given supplemental iron from the fourth month of life [4]. Dewey et al. also evaluated the effect of introducing complementary foods before six months of age in exclusively breastfed infants in Honduras [5]. They recommended iron drops for breastfed infants with birth weights between 2500 g and 3000 g. But McMillan et al. reported that term breastfed infants did not need supplemental iron until the birth weight tripled, which occurred at about 12 months of age [6]. Similarly Owen et al. found that infants breastfed until 20 weeks of life, had sufficient iron stores at 6 months of age [7]. Zavaleta et al. reported an interesting observation that maternal anemia did not affect breast milk iron or lactoferrin concentration at birth and during early lactation [8]. The iron status of exclusively breastfed infants up to six months of age, in relation to iron and lactoferrin levels of breast milk, has not been evaluated sufficiently. Thus the question of giving supplemental iron to all breastfed infants remains far from settled.

This prospective hospital based study was, therefore, conducted with the following aims and objectives: (i) To measure iron status [Hemoglobin (Hb), total iron binding capacity (TIBC), percent transferrin saturation (%TS), serum iron (SI), serum ferritin] at birth (cord blood), 14 weeks and 6 months and C-reactive protein (CRP) at 14 weeks and 6 months in exclusively breastfed term appropriate for gestational age (AGA) babies born to non-anemic and anemic mothers; (ii) To estimate total iron and lactoferrin in breast milk on day 1, 14 weeks and 6 months after birth; and (iii) To correlate iron status of the infants with iron and lactoferrin levels in the breast milk of their mothers at 14 weeks and 6 months of age.

## Methods

### Sample size

The sample size was calculated based on iron and lactoferrin contents of breast milk as reported by Zavaleta et al. [8], with the level of significance (α) = 0.05 and power of study (1 - β) = 0.90. The number arrived at was 43 babies in each group (anemic and non-anemic mothers). Considering a follow-up rate of around 50% at 6 months in our institution, 100 babies were recruited in each group so as to complete the follow-up of at least 50 babies until 6 months. After obtaining informed written consent from mothers, babies born by consecutive normal deliveries were divided into two groups. Group A consisted of term AGA babies of non-anemic mothers (Hb ≥ 11 g/dl) and Group B comprised of term AGA babies of anemic mothers (Hb 7 – 10.9 g/dl). The institutional ethical committee approved this study.

### Participants

The study was conducted at University College of Medical Sciences and National Institute of Immunolgy, New Delhi, India during 2003–2004. All mothers having normal antenatal history, with an uncomplicated singleton normal vaginal delivery were selected. Mothers with a history of pre-eclampsia, gestational diabetes, antepartum hemorrhage, tuberculosis and other chronic medical illnesses were excluded. Hb of the mothers was measured by automated haematology cell counter MS-9 (Melit Schloesing Laboratories) within 24 hrs of delivery. Those mothers having Hb ≥ 11 g/dl were classified as non-anemic (Group A) and Hb 7 – 10.9 g/dl as anemic (Group B) respectively. About 80% of mothers were from Delhi and the rest were from adjoining states. All mothers belonged to lower and middle income groups.

In both groups, term infants (gestational age 37 – 41 completed weeks) who weighed ≥ 2500 g and had an Apgar score of 8 or more at 1 minute with no gross congenital malformations, were enrolled. Breastfeeding was initiated within 30 – 60 minutes after birth. All mothers were counseled about exclusive breastfeeding and hospital follow-up at 6, 10 and 14 weeks and 6 months. At each follow-up, the babies were immunized as per Delhi State Immunization Schedule and their anthropometric parameters were recorded. The importance of exclusive breastfeeding was reinforced to mothers at each follow-up visit. Those infants who received water, non-nutritive feeds or vitamin drops during follow-up were included in the study. Infants who were fed non-human milk daily for 3 or more days with or without breast-feeds, given iron supplements and Hb < 7 g/dl were excluded. All mothers were supplemented with iron and folic acid according to the attending obstetrician.

### Sample collection and follow-up

#### Collection of cord blood samples

Immediately after delivery, 6 ml cord blood was collected in an iron-free test tube and 2 ml in a plain vial, without milking the cord, from which serum was separated and stored at -20°C. Another cord blood sample of 2 ml was taken in a vial containing ethylenediamine tetra acetic acid (EDTA) and Hb was estimated by automated haematology cell counter MS-9 (Melit Schloesing Laboratories).

#### Collection of blood sample from babies on follow-up

Five ml blood was collected by venipuncture; 3 ml in iron free test tube and 1 ml each in EDTA vial and plain vial, at 14 weeks and 6 months. Serum was separated and stored at -20°C. Hb was estimated in the EDTA vial blood sample as described above. The serum samples obtained at birth from cord blood and during follow-up from venipuncture were thawed to room temperature and TIBC, %TS and iron were estimated as per the modified guidelines of International Committee for Standardization in Haematology [9,10]. Serum ferritin was measured by enzyme linked immunosorbent assay (ELISA) (Microwell Ferritin EIA, Syntron Bioresearch Inc. Carlsbad, CA, USA). CRP was measured by latex agglutination semi quantitative method (RHELAX CRP kits, Tulip Diagnostics (P) Ltd., Goa, India). CRP < 0.6 mg/l was taken as normal and considered to be evidence of no bacterial infection in the baby.

#### Collection and analysis of breast milk

Breast milk was collected and analyzed for iron and lactoferrin on day 1, 14 weeks and 6 months after delivery as detailed by Shashiraj et al. [11]. Manual expression of breast milk was demonstrated to the mother on a model and she was requested to collect the breast milk by manual expression. After collecting around 10 ml of foremilk in a sterile acid-washed and rinsed iron-free container, the baby was breastfed for about 15 minutes and thereafter, an equal volume of hind-milk from the same breast was collected. After mixing thoroughly, the samples were kept frozen at -20°C until analyzed. Breast milk samples were thawed and mixed thoroughly before analysis. Milk was digested in concentrated nitric acid and iron was estimated by atomic absorption spectrometry [12]. For lactoferrin estimation, milk fat was removed by centrifugation at 15 000 × g for 30 minutes and lactoferrin levels were measured by ELISA method in the skimmed milk (kit supplied by Calbiochem, San Diego, CA, USA, Catalog number 427275). The breast milk lactoferrin could be measured only in 13 mothers each in both groups due to logistic constraints. One ELISA kit could support measurement of 96 samples. Lactoferrin was estimated in 78 breast milk samples and remainder were used for controls.

### Statistical analysis

Statistical analyses were performed by using SPSS Software Package (version 11.0; SPSS Inc, Chicago, IL). Mean ± standard deviation (SD) is reported unless otherwise stated. Changes in iron status variables were analyzed by using repeated measure analysis of variance followed by Tukey's honestly significant difference post hoc tests. A significance level of 0.05 was used to determine statistical significance of the observed differences adjusted for multicomparisons. Pearson's product moment correlation coefficient was used to assess correlation between iron status variables and breast milk iron and lactoferrin.

## Results

In this prospective study, the mean weight, length and head circumference of the babies of non-anemic mothers at birth were 2.84 ± 0.25 kg, 49.5 ± 0.5 cm and 34.7 ± 0.4 cm respectively. The mean weight, length and head circumference of the babies of anemic mothers at birth were 2.82 ± 0.24 kg, 49.5 ± 0.6 cm and 34.5 ± 1.1 cm respectively; the groups were similar (p > 0.05). Likewise, the mean weight (44.41 kg and 45.81 kg) and height (145.4 cm and 146.9 cm) of non-anemic and anemic mothers were also similar. All mothers belonged to lower and middle-income groups and were similar in terms of level of education and other socio-demographic factors.

In group A, 68 babies (male (M)/female (F): 36/32) at 14 weeks and 52 babies (M/F: 28/24) at 6 months were followed. In group B, 61 (M/F: 30/31) and 50 babies (M/F: 25/25) could be followed at 14 weeks and 6 months respectively. There was no significant difference in the iron parameters at birth, between the babies born to non-anemic mothers, who were lost to follow-up, compared to the babies who were followed until 6 months. However, the cord Hb of babies born to anemic mothers, who were lost to follow-up (17.48 ± 1.07 g/dl), was significantly higher (p = 0.007) than that of followed-up babies (16.59 ± 1.80 g/dl) although Hb levels were within normal limits. Similarly TIBC in babies of anemic mothers was significantly higher (p = 0.001) in the group followed-up (Table 1).

**Table 1:** Comparison of mean haemoglobin (Hb), serum iron (SI), total iron binding capacity (TIBC) and percent transferrin saturation (%TS) in cord blood of babies followed until 6 months and lost to follow-up

	Group	Followed group* Mean ± SD	Lost to follow-up Mean ± SD	p value
Hb (g/dl)	A	17.4 ± 1.6	17.1 ± 1.7	0.06
	B	16.6 ± 1.8	17.4 ± 1.1	0.01
SI (μg/dl)	A	139.8 ± 28.8	139.0 ± 27.1	0.73
	B	144.2 ± 36.5	141.4 ± 27.5	0.69
TIBC (μg/dl)	A	271.0 ± 46.8	286.2 ± 41.0	0.10
	B	281.0 ± 49.2	251.2 ± 32.7	0.01
%TS	A	57.0 ± 10.8	60.5 ± 8.8	0.27
	B	56.7 ± 11.4	56.6 ± 5.8	0.69

*Group A (babies of non-anemic mothers), n = 52 babies followed until 6 months.

Group B (babies of anemic mothers), n = 50 babies followed until 6 months

The mean Hb and serum ferritin in the babies of group A (17.43 ± 1.65 g/dl, 132.79 ± 15.19 ng/ml) and group B (16.59 ± 1.82 g/dl, 133.62 ± 9.05 ng/ml); all values were within normal limits. A significant decline was observed in these parameters in both groups at 14 weeks and 6 months (Hb decline in babies of group B was not significant between 14 weeks and 6 months) but these remained within normal limits (Table 2). At 14 weeks, eight babies had Hb < 10.5 g/dl. One of them was born to a non-anemic mother (Hb: 10.4 g/dl, serum ferritin: 41 ng/ml). Seven babies were of anemic mothers and had Hb in the range of 8.9 – 10.4 g/dl (mean serum ferritin: 56.50 ± 7.04 ng/ml). The growth of babies born to non-anemic and anemic mothers at 6 months of age, as assessed by mean gain, in weight (4.09 ± 0.17 kg vs 4.23 ± 0.22 kg), length (18.4 ± 0.79 cm vs 18.2 ± 0.66 cm) and head circumference (7.5 ± 0.38 cm vs 7.9 ± 0.41 cm) was within normal limits.

**Table 2:** Distribution of mean haemoglobin (Hb), serum iron (SI), total iron binding capacity (TIBC), percent transferrin saturation (%TS) and serum ferritin of babies in cord blood, 14 weeks and 6 months after delivery

	Group	Cord blood	p value*	14 weeks^#^	p value^†^	6 months**
Hb (g/dl)	A	17.4 ± 1.6	<0.01	12.5 ± 0.6	0.01	11.5 ± 0.5
	B	16.6 ± 1.8	<0.01	11.6 ± 0.9	0.05	11.2 ± 0.5
SI (μg/dl)	A	139.8 ± 28.8	<0.01	88.3 ± 12.2	<0.01	70.8 ± 7.2
	B	144.2 ± 36.5	<0.01	82.5 ± 13.1	<0.01	65.70 ± 9.0
TIBC (μg/dl)	A	271.0 ± 46.8	<0.01	308.8 ± 36.8	0.08	321.3 ± 34.1
	B	281.0 ± 49.2	<0.01	307.2 ± 37.4	<0.01	333.8 ± 29.3
%TS	A	57.0 ± 10.8	<0.01	28.9 ± 4.0	<0.01	22.3 ± 3.1
	B	56.7 ± 11.4	<0.01	27.5 ± 6.0	<0.01	19.8 ± 2.8
Serum ferritin (ng/ml)	A	132.8 ± 15.2	<0.01	54.6 ± 9.7	<0.01	17.8 ± 6.5
	B	133.6 ± 9.0	<0.01	55.0 ± 8.5	<0.01	17.7 ± 6.4

All values are mean ± SD.

^# ^n = 68 (Group A), n = 61 (Group B).

**n = 52 (Group A), n = 50 (Group B).

*p value within each group between day 1 and 14 weeks.

^†^p value within each group between 14 weeks and 6 months.

Iron and lactoferrin in the breast milk of non-anemic and anemic mothers decreased progressively from day 1 to 14 weeks and up to 6 months and the difference was significant (p < 0.001). But, no significant difference (p > 0.05) was noted in these parameters between non-anemic and anemic mothers at any postpartum age (Table 3).

**Table 3:** Distribution of breast milk iron (mg/l) and lactoferrin (mg/ml) on day 1, 14 weeks and 6 months in non-anemic (group A) and anemic mothers (group B)

**Breast Milk**	**Mothers**	**Day 1 **	p value*	**14 weeks**^#^	**p value**^†^	6 months**
		**Mean ± SD**		**Mean ± SD**		**Mean ± SD**
Iron (mg/l)	Group A	0.89 ± 0.13	<0.01	0.34 ± 0.04	<0.01	0.26 ± 0.04
	Group B	0.86 ± 0.14	<0.01	0.33 ± 0.05	<0.01	0.27 ± 0.04
Lactoferrin (mg/ml) (n = 13)	Group A	12.02 ± 2.58	<0.01	5.84 ± 1.47	0.98	5.85 ± 1.09
	Group B	12.91 ± 2.83	<0.01	6.68 ± 1.17	0.53	6.37 ± 1.33

All values are mean ± SD.

* Comparison within each group between day 1 and 14 weeks.

† Comparison within each group between 14 weeks and 6 months.

# n = 68 (Group A), n = 61 (Group B).

** n = 52 (Group A), n = 50 (Group B).

The declining Hb, SI and serum ferritin in the babies of non-anemic and anemic mothers significantly correlated with the declining breast milk iron and lactoferrin concentration. However, at any given point of time, the breast milk iron and lactoferrin did not correlate with the Hb and other iron parameters of the babies (Table 4). CRP was measured as a marker of infection and to detect its confounding effect on serum ferritin level. The concentrations of CRP in babies of both groups at 14 weeks and 6 months were normal.

**Table 4:** Correlation of haemoglobin (Hb), serum iron (SI) and serum ferritin (Sf) of babies in both groups with breast milk iron (BMi) and breast milk lactoferrin (BML)

**Variable**		**Group A**		**Group B**
	**r-value**	**Regression equation**	**r-value**	**Regression equation**
Hb vs BMi*	0.863	Hb = 9.659+8.236 × BMi	0.876	Hb = 8.971+8.578 × BMi
SI vs BMi*	0.808	SI = 50.511+100.496 × BMi	0.802	SI = 40.637+119.438 × BMi
Sf vs BMi*	0.912	Sf = -9.932+159.375 × BMi	0.910	Sf = -9.032+161.766 × BMi
Hb vs BML*	0.812	Hb = 8.825+0.607 × BML	0.772	Hb = 8.485+0.489 × BML
SI vs BML*	0.793	SI = 26.205+9.087 × BML	0.680	SI = 21.473+8.766 × BML
Sf vs BML*	0.795	Sf = -22.825+11.207 × BML	0.811	Sf = -32.277+11.691 × BML

*p-value < 0.001 (simple regression analysis).

Group A- non-anemic mothers and their babies.

Group B- anemic mothers and their babies.

## Discussion

Domelloff et al. were the first to report the reference values for iron status variables in exclusively breastfed infants [13]. They reported Hb >10.5 g/dl as normal 2 SD cut-off at four and six months of age. The 2 SD cut-offs for serum ferritin were < 20 ng/ml and < 9 ng/ml at these ages respectively. In the present study, none of the babies in either group were found to be iron deficient at 14 weeks and 6 months although at 14 weeks of life eight babies were anemic as per these criteria [13]. Their serum ferritin was >41 ng/ml which signified that iron stores were normal. The Hb of all these babies increased to non-anemic ranges (>10.5 g/dl) with normal age-specific serum ferritin levels at six months. The low Hb level in these babies at 14 weeks was not due to iron deficiency but perhaps was a result of delayed onset of haematopoiesis compared to their peers [3]. Once the trigger to start Hb formation was activated these babies produced enough Hb later on, and were able to overcome their earlier deficiency.

Similarly at six months of age, none of the babies in either group were iron-deficient or anemic with a lone exception. In one baby of anemic mother, who had normal Hb level at 14 weeks, the Hb level fell just below 10.5 g/dl (10.4 g/dl) with age-normal serum ferritin level (11 ng/ml) which indicated that the baby was not iron deficient [13]. Our observations regarding Hb, SI, TIBC, %TS and serum ferritin of babies in both groups, at six months of age, were in concordance with other studies [14-16].

Pisacane et al. studied the iron status of 30 infants who had been breastfed until their first birthday and who had never received cow milk, medical iron or iron-enriched formula and cereals [17]. None of the infants who were exclusively breastfed for seven months or more and 43% of those who were breastfed for a shorter duration, were anemic (Hb < 11 g/dl) at 12 months of age. The duration of exclusive breastfeeding was significantly longer among non-anemic infants (6.5 vs 5.5 months) [17]. Murray et al. studied the effect of iron status of Nigerian mothers on the concentration of iron in breast milk and reported that, infants feeding entirely on breast milk appeared to have normal iron status at six months [14]. Duncan et al. followed 33 exclusively breastfed infants from birth to six months for their iron status [15]. They concluded that infants who were exclusively breastfed for the first six months of life were not at a higher risk for the development of iron deficiency anemia or the depletion of iron stores during that time [15].

In another study by Lonnerdal and Hernall of exclusively breastfed infants and infants fed cow's milk based formula containing either 4 mg iron/l or 7 mg iron/l, there were no significant differences in the haematological indices among the groups at 6 months age and the iron status of the infants was satisfactory [16]. They also found that the concentration of serum transferrin receptors was highest in breastfed infants, and lowest in the infants who received high concentration of iron (7 mg/l) [16]. This finding suggested that breastfed infants were probably on the verge of becoming iron deficient, although their serum ferritin levels and other haematological indices were normal. Interestingly, in the above study, the normal haematological values used for comparison were obtained from a selected group of healthy term infants (> 3 kg birth weight) who were receiving continuous iron supplementation during the first year of life [18,19], but still breastfed infants were not seen doing badly.

As we have reported previously, breast milk iron and lactoferrin concentration had no relationship with the mother's Hb and iron status [11]. The wide range in the breast milk iron values reported in the literature may be due in part, to differences in sampling procedures as well as stage of lactation. Iron content of human milk is highest in early transitional milk (0.97 mg/ml) but decreases steadily during lactation, reaching a level of approximately 0.35 mg/ml at 1 month of lactation to 0.20 mg/ml at 6 months [11,20-22]. In the present study from birth to six months, the mean breast milk iron ranged from 0.89 to 0.26 mg/l in the non-anemic (group A) and 0.86 to 0.33 mg/l in the anemic (group B) mothers. Mean breast milk lactoferrin levels in non-anemic mothers (group A) were 12.02 mg/ml, 5.84 mg/ml and 5.85 mg/ml respectively at day 1, 6 weeks and 6 months while in anemic mothers (group B), levels were 12.91 mg/ml, 6.68 mg/ml and 6.37 mg/ml respectively at the same time points. No significant difference was noted in the breast milk iron and lactoferrin between non-anemic and anemic mothers on day 1, 14 weeks and 6 months after delivery.

Houghton et al. studied breast milk lactoferrin levels in relation to maternal nutritional status [23]. Lactoferrin concentration was significantly higher in the first 15 days of lactation (ranging from 2.82 mg/ml to 3.49 mg/ml) than later (ranging from 0.66 mg/ml to 1.42 mg/ml). There are few other studies regarding breast milk lactoferrin concentration mainly during early lactation [8,24,25]. Lien et al. estimated lactoferrin in the breast milk of mothers from nine countries by HPLC method and reported that its level ranged from 1.37 to 2.12 g/L (mean 1.83 ± 0.67 g/L) and was significantly lower in Mexican and Australian mothers compared to Canadian, Chinese and British mothers [26]. Thus ethnic and racial factors do appear to affect lactoferrin levels in the breast milk. In the above study, Indian mothers were not included. The lactoferrin levels in our study were higher when compared to the available literature. However, lactoferrin levels in our mothers, both non-anemic and anemic, behaved in a completely different way from that reported by Lien et al. [26]. The lactoferrin levels in our mothers decreased significantly from day 1 to 14 weeks of lactation but remained fairly consistent from 14 weeks to 6 months, unlike Australian mothers, where lactoferrin levels remained fairly constant until 350 days of lactation duration, and in contrast to British mothers in whom lactoferrin declined sharply in the same period. Is the obvious difference in the breast milk lactoferrin values in our study because of the method of estimation of lactoferrin, subject variation (race/ethnicity) or a small sample size (n = 13 in each group)? It is a matter for further research. The study has conclusively showed that there was no correlation between the iron status of exclusively breastfed infants with breast milk iron and lactoferrin until six months of age.

Our findings of breast milk iron levels were in concordance with other studies [8,20,21]. Many studies in the past have assessed the iron status of exclusively breastfed infants, but unfortunately, very few have attempted to look at the relationship between breast milk iron and lactoferrin with the iron status of exclusively breastfed infants in first six months of life. It was not surprising that exclusively breastfed infants neither developed iron deficiency nor iron deficiency anemia. The breast milk iron and lactoferrin are efficiently absorbed in the gut and together with body iron stores, the iron supply is sufficient to maintain normal iron metabolism in the first six months of life in term AGA babies. Therefore, there is no need to add iron-rich foods or therapeutic iron to exclusively breastfed term infants until six months of life lest it may harm the baby [27].

## Conclusion

We found that: (i) Babies of non-anemic and anemic mothers who were exclusively breastfed until six months age did not develop iron deficiency anemia or iron deficiency; (ii) The iron status of the babies had no relation with the breast milk iron and lactoferrin concentration at any particular time; (iii) The declining haemoglobin and serum ferritin of babies of non-anemic and anemic mothers significantly correlated with the declining breast milk iron and lactoferrin concentration. Given the importance of iron nutrition during the first year of life, it was important to address the issue of iron status of exclusively breastfed babies up to six months age and its relation to the breast milk iron and lactoferrin content.

## Competing interests

The author(s) declare that they have no competing interests.

## Authors' contributions

SR was the principal investigator, collected the data and drafted the manuscript. MMAF conceptualized and supervised the study, analyzed results and reviewed final discussion and will act as guarantor. UR analyzed the hematology results. OS did breast milk analysis. All authors have read and approved the final manuscript.
